# Influence of Inflammatory Cytokines IL-1*β* and IFN*γ* on Sarcoplasmic Aggregation of p62 and TDP-43 in Myotubes

**DOI:** 10.1155/2023/9018470

**Published:** 2023-09-12

**Authors:** Bryony McCord, Richard M. Day

**Affiliations:** Centre for Precision Healthcare, UCL Division of Medicine, University College London, London WC1E 6JF, UK

## Abstract

Skeletal muscle of patients with sporadic inclusion body myositis (sIBM) presents with inflammation, including upregulation of inflammatory cytokines such as interferon *γ* (IFN*γ*). Non-inflammatory features are also observed, like the sarcoplasmic accumulation of proteins including TDP-43 and p62. This study aimed to investigate the effect of IFN*γ* and interleukin 1-*β* (IL-1*β*) on TDP-43 and p62 aggregation in vitro. Primary human myotubes were treated with IL-1*β* (20 ng/mL) and IFN*γ* (750 ng/mL) separately or combined for 48 hr. Sarcoplasmic TDP-43 aggregates and p62 puncta were assessed using image analysis for size, frequency, and colocalization with each other. Total protein expression of TDP-43, p62 and LC3 was assessed using western blotting. The subcellular localization of TDP-43 was also analyzed using image analysis. Combined IL-1*β* and IFN*γ* treatment increased puncta size of p62 compared to control (0.49 ± 0.13 *µ*m^2^ versus 0.28 ± 0.06 *µ*m^2^), without affecting puncta frequency or p62 expression but with an increased LC3II/LC3I ratio, suggesting autophagic alterations. IL-1*β* or IFN*γ* did not alter p62 puncta size or frequency, suggesting a combined insult of multiple inflammatory mediators is necessary to cause p62 alterations. IL-1*β* increased p62 protein expression in an autophagy-independent manner. None of the cytokine treatments affected TDP-43 protein expression, size, or frequency of TDP-43 aggregates or localization, suggesting IL-1*β* and IFN*γ* may influence TDP-43 processing in human skeletal muscle cells. TDP-43 was localized to the sarcoplasm under control conditions, suggesting this may not be a pathological feature. Overall, sIBM-like TDP-43/p62 features were not triggered by IL-1*β* and/or IFN*γ*.

## 1. Introduction

Sporadic inclusion body myositis (sIBM) is the most common form of myositis in people over the age of 50 [1, 2]. Symptoms of sIBM include muscle weakness, often beginning in the finger flexors and quadriceps, with an asymmetrical presentation. Patients have difficulty standing and walking, are more prone to falls, and have reduced grip strength, with muscle strength declining at a rate of between 3.5%–28% per year, depending on the assessment method used [3, 4]. Currently, there are no effective treatments for sIBM that show clinical benefit [5, 6].

At present, the cause of sIBM symptoms is unknown, and there are many mechanisms that appear dysregulated in this disease. Pathological features of sIBM can broadly be split into inflammatory or non-inflammatory. Infiltrates of mononucleated immune cells, mostly consisting of CD8+ cytotoxic T cells, can be seen in the endomysium and the muscle fibers themselves [2, 5]. A range of inflammatory cytokines are found upregulated in sIBM compared to healthy muscle or other inflammatory myopathies, including interferon *γ* (IFN*γ*), tumor necrosis factor *α* (TNF*α*), interleukin (IL)-7, and IL-32 [7].

Noninflammatory features of sIBM include rimmed vacuoles [8] and inclusion bodies, which are areas of protein aggregation within the sarcoplasm [9–11]. Over 80 different proteins have been identified as aggregated within inclusion bodies [5], and 213 proteins are upregulated within rimmed vacuoles compared to non-vacuolated sIBM muscle regions [12]. TAR DNA binding protein 43 (TDP-43) and p62 are two proteins aggregated in sIBM and have been suggested as potential biomarkers differentiating sIBM over other related inflammatory myopathies [13–17].

TDP-43 has a plethora of roles surrounding DNA and RNA processing, such as mRNA transport, stability, and splicing regulation. TDP-43 also has roles in stress sensing and cell survival by forming stress granules with halted mRNA transcripts [18]. Protein levels of phosphorylated TDP-43 and its 25 and 35 kDa truncated forms are upregulated in the skeletal muscle fibers of sIBM patients compared to healthy controls [19]. TDP-43 is usually located in the nucleus. The presence of TDP-43 within the sarcoplasm with a lack of nuclear TDP-43 has been suggested as a sensitive marker for sIBM, with its presence detected in 25% of myofibres compared to 2.8% positivity for rimmed vacuoles [15]. Furthermore, TDP-43 has been found accumulated in all biopsies of patients classified as having definite sIBM and in 31% of possible sIBM cases [13].

p62 is involved in the degradation of ubiquitinated proteins through both the ubiquitin proteasome system (UPS) and autophagic pathways [20]. In sIBM, p62 protein is increased in sIBM compared to healthy controls, and myofibres with high p62 levels show high levels of TDP-43 and mitochondrial staining [19]. However, RNA levels of p62 were decreased in sIBM patients compared to normal muscle and other myositis diseases [21]. p62 has been suggested as an sIBM marker as it can be found in 100% of definite sIBM cases and 37% of possible cases [13].

Inflammatory cytokines are upregulated in sIBM. Notably, IFN*γ* transcripts are elevated in sIBM compared to control and other inflammatory myopathies [7, 22]. In myofibres attacked by CD8+ T cells, upregulation of IFN*γ* receptor 2 (IFNGR2) was observed compared to healthy or non-attacked myofibres. The amount of IFNGR2 upregulation in skeletal muscle was partially correlated with the number of attacking CD8+ cells [23]. Transcription of the guanylate-binding protein 6 (GBP6) gene, which is induced by IFN*γ* signaling [24], was seven times higher in sIBM compared to controls or other inflammatory myopathies [25].

Previous work has used in vitro treatment of myotubes with inflammatory cytokines IL-1*β* and IFN*γ* to examine pathological features related to sIBM, such as TDP-43 aggregation and mislocalization in rat myotubes [9], *β* amyloid aggregation [26], and other degenerative features [27–29]. However, the effect of IL-1*β* and IFN*γ* on TDP-43 and p62 in human myotubes has not previously been investigated. The aim of this study was to further investigate the roles of IL-1*β* and IFN*γ* on the sIBM-like pathological features of TDP-43 and p62 aggregation as well as TDP-43 mislocalization in human myotubes, with the hypothesis that IL-1*β* and/or IFN*γ* treatment triggers these non-inflammatory features.

## 2. Materials and Methods

All materials were purchased from ThermoFisher Scientific, UK, unless otherwise stated.

### 2.1. Cell Culture

Skeletal muscle-derived cells from Lonza Clonetics (UK CC-2580) and Cook MyoSite (USA, SK-1111) were grown in either Skeletal Muscle Growth Media (Promocell, Germany) with 1% penicillin-streptomycin and 10% fetal bovine serum (FBS) or Ham's F10 nutrient mix supplemented with 20% FBS, 1 *µ*M dexamethasone (Sigma, UK), 10 ng/mL human basic fibroblast growth factor (bFGF, Peprotech, UK), and 1% penicillin-streptomycin. After 48 hr in growth media, 4 × 10^4^ cells in 24 well plates were differentiated in N2 differentiation medium (DMEM/F12 1 : 1, 1% N2 supplement, 1% L-glutamine, 1% insulin-transferrin-selenium, and 0.2% penicillin-streptomycin) for 7 days. Cells were maintained at 37°C with 5% CO_2_. Most experiments were conducted at passage 7. For cytokine treatment, 20 ng/mL IL-1*β* or 750 ng/mL IFN*γ* (Peprotech, UK) separately or combined was added to differentiation media and incubated for 48 hr. Control samples consisted of the addition of no cytokine. Different myogenic donors were used for different experiments.

### 2.2. Cytotoxicity Assay

The cytotoxic effect of cytokine treatment was measured using the CytoTox 96® non-radioactive cytotoxicity assay (Promega, UK). Myotubes were differentiated for 7 days before 48 hr incubation with the cytokines. Results were normalized to a positive lysis control where cells were incubated with lysis solution for 45 min before commencing the assay.

### 2.3. Immunofluorescent Staining and Microscopy

4 × 10^4^ skeletal muscle cells were seeded onto Gibco Geltrex-coated 13 mm round coverslips (VWR, UK) in 24 well plates. After differentiation, cells were fixed with 4% formaldehyde solution for 10 min and permeabilized in 0.1% triton X 100 for 15 min, then blocked with 5% goat serum for up to 1 hr. Primary antibodies were incubated by inverting the coverslip onto a 30 *µ*L drop of antibody solution on Parafilm overnight at 4°C (see Table S1 in the Supplementary Material for comprehensive details of the antibodies and the dilutions used). Alexafluor-conjugated secondary antibodies were added for 1 hr at room temperature. HCS CellMask deep red or red was added at 1 : 5,000 for 30 min followed by 5 *µ*g/mL 4′, 6-diamidino-2-phenylindole (DAPI) counterstaining. Coverslips were mounted onto glass slides with DAKO fluorescence mounting medium (Agilent, USA) and imaged using either an Olympus IX81-ZDC inverted widefield microscope or a Zeiss LSM 980 Airyscan 2 confocal microscope.

### 2.4. Image Analysis

#### 2.4.1. p62 Sarcoplasmic Puncta Analysis

Four *z*-stacks composed of 6 images each with a voxel depth of 0.3 *µ*m were obtained using confocal microscopy for each treatment condition. p62 particle frequency was determined using NIH Image J using the “analyse particles” function with a pixel size of over 2 for each slice of the *z*-stack. p62 particle frequencies were normalized to percentage area coverage per image using HCS CellMask staining to account for differences in myotube size.

#### 2.4.2. TDP-43 Sarcoplasmic Aggregate Analysis

The same samples for p62 analysis were used for TDP-43 sarcoplasmic aggregate analysis. Sarcoplasmic aggregates were identified manually in 3 *z*-stacks slices as punctate areas of staining with higher intensity than surrounding sarcoplasmic staining. Using NIH Image J, freehand regions were drawn around aggregates to measure their area in *µ*m^2^. TDP-43 aggregate frequencies were normalized to the percentage area coverage of the cell mask area. TDP-43 co-localization with p62 was assessed for each identified TDP-43 aggregate. Any area of overlap between TDP-43 and p62 objects over 2 pixels was considered co-localized.

#### 2.4.3. TDP-43 Localization

Fluorescent images were obtained using an Olympus IX81-ZDC inverted widefield microscope. TDP-43 was classified as being within the nucleus, sarcoplasm, nucleus and sarcoplasm, or neither (not expressed) by manual identification. TDP-43 localization was classified for all visible cells, both multinucleated and single-nucleated cells. A multinucleated cell was classed as having nuclear (or nuclear and sarcoplasmic) staining if one or more nuclei showed clear TDP-43 expression. The median number of cells per image in all conditions was 44. All image analysis for TDP-43 and p62 was conducted blind.

### 2.5. SDS-PAGE and Western Blotting

Approximately 2.4 × 10^5^ skeletal muscle-derived cells were lysed in RIPA buffer (Sigma–Aldrich, UK) with Pierce™ protease and phosphatase inhibitors. The protein concentration of lysates was determined using a Micro Lowry kit with Peterson's modification (Sigma, UK). Approximately 10 *µ*g of protein was denatured at 95°C for 5 min in Bolt SDS (sodium dodecyl sulfate) sample buffer and Bolt reducing agent. PAGE (polyacrylamide gel electrophoresis) separation was performed with Bolt 4%–12% Bis-Tris mini protein gels at 200 V, followed by Western blotting onto PVDF membranes at 30 V. Blots were blocked in 5% milk-TBS-T, incubated with primary antibodies overnight at 4°C, and secondary antibodies for 1 hr at room temperature (Table S1). Enhanced chemiluminescence (ECL) reagent was added for 1 min before film development using an SRX-101A processor (Konica Minolta, UK). Densitometry was performed on scanned film images using NIH Image J, and proteins of interest normalized to glyceraldehyde 3-phosphate dehydrogenase (GAPDH) loading control. For re-probing, membranes were stripped using Restore™ Western blot Stripping Buffer for up to 1 hr at room temperature with shaking followed by up to 40 min at 37°C. Membranes were washed and re-blocked with 5% milk-TBS-T, re-probed with secondary antibodies, and re-exposed to check for primary antibody removal.

### 2.6. Statistical Analysis

Graph plotting and statistics were performed using GraphPad Prism 7. The Gaussian distribution of the data was tested using Shapiro–Wilk test, and appropriate statistical analyses for each data set are listed in the Results section. Results are shown as mean ± standard error of mean (SEM) for normally distributed data or median ± interquartile range for non-normally distributed data.

## 3. Results

### 3.1. Cytokine Treatments Were Not Cytotoxic to Myotubes

**Figure 1:**
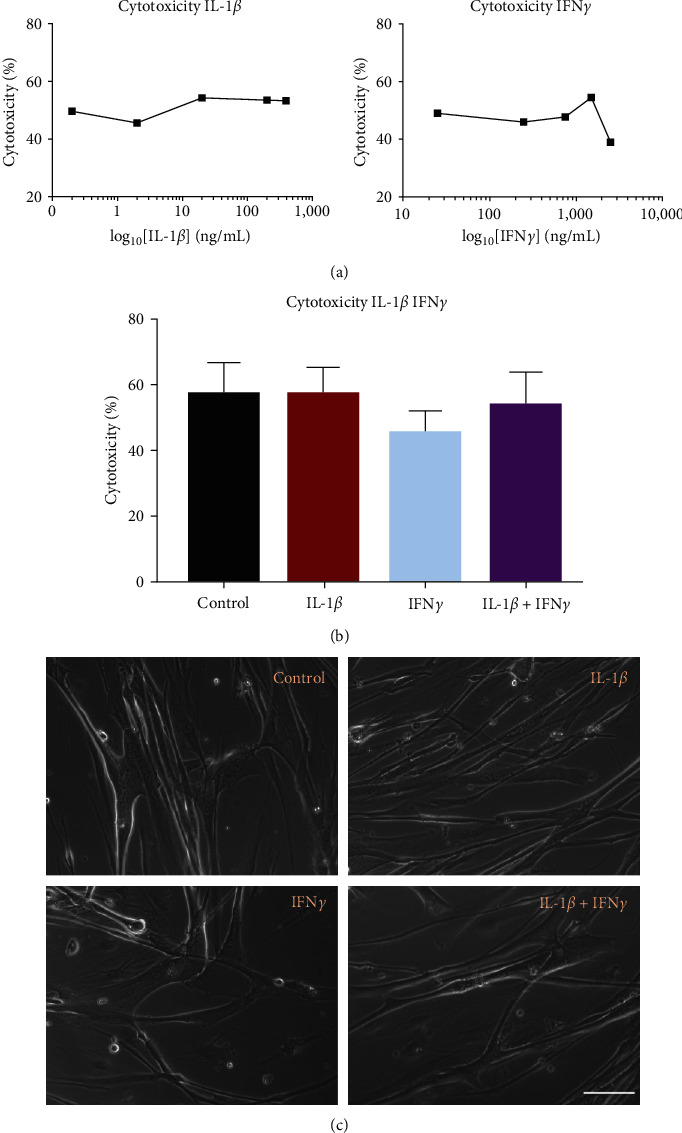
Cytotoxicity of IL-1*β* and IFN*γ* alone or in combination. (a) Multiple concentrations of IL-1*β* and IFN*γ* were tested for cytotoxicity on one donor. (b) Cytotoxicity of IL-1*β* and IFN*γ* separately or combined was measured 48 hr after adding to differentiated myotubes. There was no difference between control and IL-1*β*, control and IFN*γ*, or control and IL-1*β* + IFN*γ* treated myotubes. *n* = 6 myogenic donors. (c) There was no obvious morphological difference in myotubes between the control and treatment conditions under light microscopy. Scale bar = 100 *μ*m.

To ensure that IL-1*β* and IFN*γ* treatments were not causing excessive cell death in cultured myotubes, the cytotoxicity of cytokine treatments was measured via lactate dehydrogenase release after 48 hr of treatment. Firstly, a range of concentrations of each cytokine were tested in a preliminary experiment on one myogenic donor with 5 days of differentiation and 48 hr of cytokine treatment (Figure 1(a)), showing no trend in cytotoxicity with increasing IL-1*β* or IFN*γ* concentration. IL-1*β* concentrations tested were 0.2, 2, 20, 200, and 400 ng/mL. IFN*γ* concentrations tested were 25, 250, 750, 1,500, and 2,500 ng/mL. IL-1*β*, IFN*γ*, or combined treatment was tested for cytotoxicity on 6 myogenic donors at IL-1*β* 20 ng/mL and IFN*γ* 750 ng/mL. Neither IL-1*β* nor IFN*γ* nor combination treatment significantly affected cytotoxicity compared to untreated myotubes (Figure 1(b)). Furthermore, under light microscopy, there were no morphological alterations with any cytokine treatments (Figure 1(c)).

### 3.2. TDP-43 Localization Was Not Affected by IL-1*β* + IFN*γ* Cytokine Treatment

**Figure 2:**
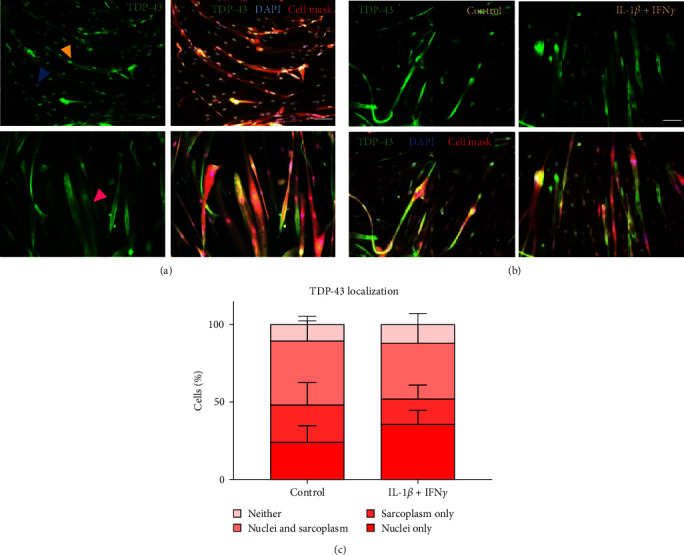
TDP-43 localization with IL-1*β* + IFN*γ* treatment. (a) Images of TDP-43 distribution within untreated control myotubes, examples of cells with a nuclear only (blue arrowhead) or nuclear and sarcoplasmic (yellow arrowhead) TDP-43 distribution. Example of sarcoplasmic only (pink arrowhead) TDP-43 distribution. scale bar = 100 *µ*m. (b) immunofluorescent images of TDP-43 localization in control and IL-1*β* + IFN*γ* treated myotubes. scale bar = 100 *µ*m. (c) There was no difference between the control and IL-1*β* + IFN*γ* cytokine groups for TDP-43 distribution via two-way ANOVA. *n* = 5 myogenic donors.

Previously, TDP-43 mislocalization to the sarcoplasm and not the nucleus has been shown in sIBM muscle [15] and with IL-1*β* treatment [9]. Here, the effect of IL-1*β* and IFN*γ* combined on TDP-43 localization was examined by classifying the presence of TDP-43 in each cell as either nuclear, sarcoplasmic, nuclear and sarcoplasmic, or neither/low expression not detectable via immunofluorescence (Figure 2(a)). Even in the control group, cells presented with sarcoplasmic only TDP-43 staining (Figure 2(a)–2(c)). TDP-43 localization was not altered by treatment with IL-1*β* + IFN*γ* (Figure 2(c)), as determined by a two-way ANOVA. There was no difference in the distribution of TDP-43 between the subcellular compartments as assessed via a two-way ANOVA, suggesting TDP-43 is spread evenly between these locations.

### 3.3. TDP-43 Aggregates Were Not Affected by IL-1*β* + IFN*γ* Treatment but p62 Puncta Size Was Increased

**Figure 3:**
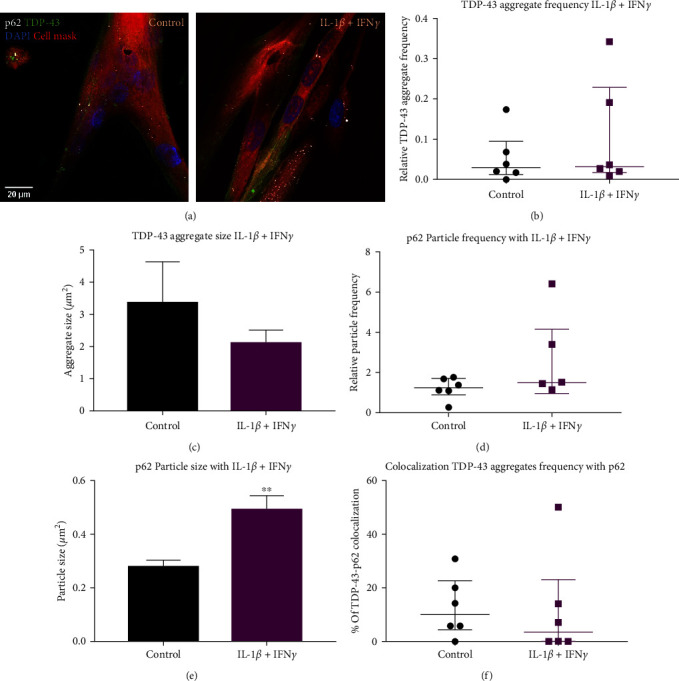
Sarcoplasmic TDP-43 aggregates and p62 puncta with IL-1*β* + IFN*γ*. (a) Representative images of TDP-43 and p62 within control and IL-1*β* + IFN*γ*-treated myotubes. There was no difference between control or IL-1*β* + IFN*γ* for TDP-43 aggregate frequency relative to cell area (b) or aggregate size (c). Relative p62 puncta frequency was not different between control and IL-1*β* + IFN*γ* treatment (d), however, with IL-1*β* + IFN*γ* p62 puncta were significantly larger (*p*=0.0046) (e). There was no difference in colocalization of TDP-43 aggregates with p62 puncta between control and IL-1*β* + IFN*γ* (f). Student's *T*-test or Mann–Whitney *U* test. *n* = 6 myogenic donors.

TDP-43 sarcoplasmic aggregates were assessed using image quantification. Not all images contained TDP-43 aggregates, and aggregates were observed in the control group (Figure 3(a)). The likelihood of an image containing TDP-43 aggregates was not affected by combined IL-1*β* and IFN*γ* cytokine treatment compared to the untreated control (Fisher's exact test, *p*=0.341). TDP-43 aggregate frequency (Figure 3(b)) or aggregate size (Figure 3(c)) were not affected by IL-1*β* + IFN*γ* treatment. The mean TDP-43 aggregate size in the control group was 3.35 ± 1.28 *µ*m^2^.

Sarcoplasmic p62 displayed small puncta, and all myogenic cells examined showed p62 expression. p62 was also observable in some nuclei and with weak diffuse staining in the sarcoplasm (Figure 3(a)). The relative frequency of p62 puncta was not affected by IL-1*β* + IFN*γ* compared to the control (Figure 3(d)). However, IL-1*β* + IFN*γ* treatment caused an increase in p62 puncta size compared to control (Figure 3(e)). The mean size of p62 puncta in the control group was 0.28 ± 0.06 *µ*m^2^, and in the IL-1*β* + IFN*γ* treatment group was 0.49 ± 0.13 *µ*m^2^.

Some TDP-43 aggregates were observed colocalized with p62 puncta. Any colocalization was mostly partial, with some of the TDP-43 aggregate area also showing p62 puncta staining. There was no difference in the percentage of TDP-43 aggregates that colocalized with p62 puncta between the control and IL-1*β* + IFN*γ* treatment groups (Figure 3(f)). The median percentage of TDP-43 aggregates that colocalized with p62 puncta was 10 ± 18%.

After finding that p62 size was increased by combined IL-1*β* and IFN*γ* treatment, the effects of these two cytokines as individual treatments were tested to examine if only IL-1*β* or IFN*γ* were responsible for this effect. The influence of IL-1*β* or IFN*γ* was also tested for effects on TDP-43. Due to limited donor samples, three of the same myogenic donors that were used for IL-1*β* + IFN*γ* analysis were used for individual cytokine treatments, and two donors that were not used for IL-1*β* + IFN*γ* analysis were assessed (see Table S3). To ensure a level of consistency between the two sets of experiments with different donors, the control condition for the IL-1*β* + IFN*γ* and the IL-1*β* or IFN*γ* experiments were compared, showing no statistical difference in any measurement (Table S2).

### 3.4. TDP-43 Localisation Was Not Affected by IL-1*β* or IFN*γ*

**Figure 4:**
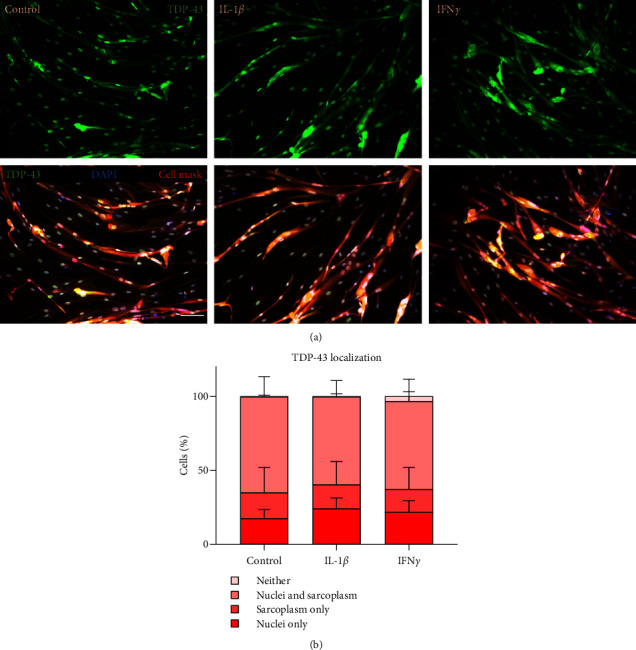
TDP-43 localization with IL-1*β* or IFN*γ* treatment. (a) Immunofluorescent images of TDP-43 localization in untreated control, IL-1*β*, or IFN*γ*-treated myotubes. (b) There was no difference between control-treated myotubes and those treated with IL-1*β* or IFN*γ* treatments for TDP-43 subcellular localization. *n* = 5 myogenic donors.

Widefield microscopy was used to investigate whether IL-1*β* or IFN*γ* individual treatments affected TDP-43 localization (Figure 4(a)). There was no difference between the control group and either IL-1*β* or IFN*γ* for the distribution of TDP-43 within the subcellular compartments (Figure 4(b)). The distribution of TDP-43 between the subcellular compartments regardless of treatment condition was different (two-way ANOVA *p* < 0.0001). The subcellular localization that showed the highest percentage of TDP-43 was nuclei and sarcoplasm.

### 3.5. TDP-43 Aggregates or p62 Puncta Were Not Affected by IL-1*β* or IFN*γ* Treatment

**Figure 5:**
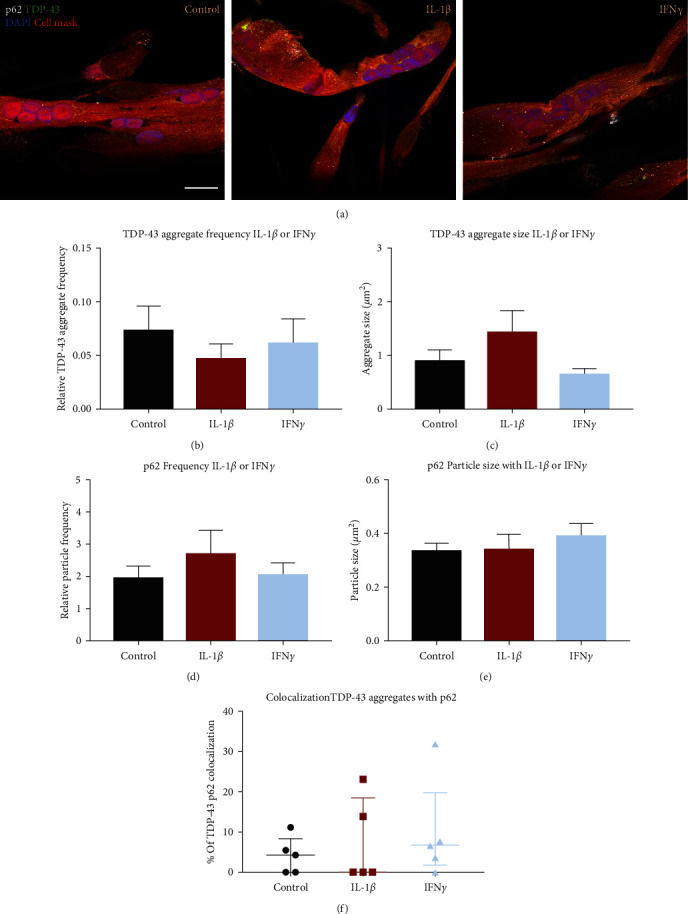
Sarcoplasmic TDP-43 aggregation and p62 puncta with IL-1*β* or IFN*γ*. (a) Representative images of TDP-43 and p62 distribution in control, IL-1*β*, or IFN*γ*-treated myotubes. There was no difference between control and IL-1*β* or control and IFN*γ* for TDP-43 aggregate frequency relative to cell area (b) or aggregate size (b). p62 relative particle frequency (d) or size (e) was not different between control and IL-1*β* or control and IFN*γ*. The percentage of TDP-43 aggregates that colocalized with p62 puncta was not different between control and IL-1*β* or control and IFN*γ* (f). One-way ANOVA or Krustal–Walis test. *n* = 5 myogenic donors.

The effect of IL-1*β* or IFN*γ* treatment on TDP-43 sarcoplasmic aggregation was also assessed to determine single cytokine effects (Figure 5(a)). The likelihood of an image containing TDP-43 aggregates was not affected by either cytokine treatment (*χ*^2^*p*=0.392). The frequency of TDP-43 sarcoplasmic aggregates relative to cell area was not affected by any cytokine treatment (Figure 5(b)). TDP-43 aggregate size was not affected by IFN*γ* or IL-1*β* (Figure 5(c)). However, 3 of 5 examined myogenic donors showed increased TDP-43 aggregate size with IL-1*β* compared to control (see Figure S1 in the Supplementary Material). The mean TDP-43 sarcoplasmic aggregate size in the control group was 0.89 ± 0.21 *µ*m^2^.

p62 displayed similarly to those in the IL-1*β* + IFN*γ* treatment group, with small puncta, some diffuse sarcoplasmic staining, and some nuclear puncta. (Figure 5(a)). There was no difference in p62 puncta frequency relative to cell area between the control group and either cytokine treatment group (Figure 5(d)). There was also no difference between any treatment conditions for the size of the p62 puncta (Figure 5(e)). The mean size of p62 puncta in the control group was 0.33 ± 0.03 *µ*m^2^.

Similarly to the combined IL-1*β* + IFN*γ* treatment condition, some TDP-43 sarcoplasmic aggregates colocalized with p62 puncta, with mostly partial colocalization. There was no difference between the control and either IL-1*β* or IFN*γ* treatments for the percentage of TDP-43 aggregates that colocalized with p62 puncta, with a median colocalization of 4% ± 8%.

### 3.6. p62 Expression Was Increased with IL-1*β* Treatment and LC3 Expression Was Affected by IL-1*β* + IFN*γ* Treatment

**Figure 6:**
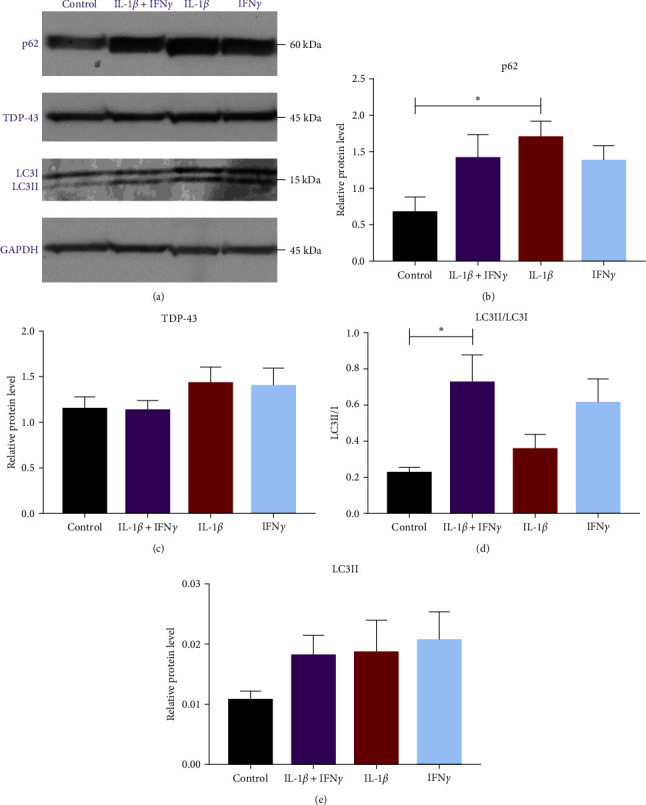
p62 expression was increased with IL-1*β* and LC3II/LC3I ratio increased with IL-1*β* + IFN*γ* treatment. (a) Western blot of p62, TDP-43, and LC3 with GAPDH loading control. (b) p62 densitometry normalized to GAPDH. There was an increase compared to control for p62 protein expression with IL-1*β* treatment (*p*=0.0223), but no difference between control and the other cytokine treatments. (c) Densitometry analysis of TDP-43 protein expression normalized to GAPDH showed no difference in TDP-43 expression between any of the conditions. (d) Ratio of LC3II to LC3I expression. IL-1*β* + IFN*γ* increased LC3II/LC3I ratio compared to the control (*p*=0.0116). There was no difference compared to the control for IL-1*β*, or IFN*γ*. (e) Total LC3II relative to GAPDH. There was no difference between any of the conditions (one-way ANOVA *p*=0.352). One-way ANOVA with Dunnett's multiple comparisons. *n* = 6 myogenic donors.

Following the discovery that IL-1*β* + IFN*γ* treatment caused increased p62 puncta size but not IL-1*β* or IFN*γ* individually, the effect of these cytokines was assessed for p62, TDP-43, and LC3 protein expression using western blotting (Figure 6(a)). GAPDH was used as a loading control, and protein expression was normalized to GAPDH (Figures 6(b), 2(c), and 2(e)). Densitometry analysis showed that compared to the control, p62 expression was increased with IL-1*β* treatment but not with IFN*γ* or IL-1*β* + IFN*γ* treatment (Figure 6(b)). There was no difference in TDP-43 expression between the control and any cytokine treatment group (Figure 6(c)).

LC3 protein expression was examined to understand the autophagic activity of cells under each treatment condition. Both p62 and LC3 are involved in autophagy, with an increase in LC3II and a decrease in p62 expression associated with increased autophagic flux [30]. Compared to control, myogenic cells treated with IL-1*β* + IFN*γ* showed an increased LC3II/LC3I ratio, while IL-1*β* or IFN*γ* showed no difference. The mean LC3II/LC3I ratio in control was 0.22 ± 0.04 and in IL-1*β* + IFN*γ* was 0.72 ± 0.15, a 3.2-fold increase. All LC3II/LC3I ratios were below 1, showing higher expression of LC3I than LC3II (Figure 6(d)). However, when assessing LC3II expression normalized to GAPDH, there was no difference between any of the conditions (Figure 6(e)).

## 4. Discussion

There are many pathological features of sIBM that can be categorized as either inflammatory or non-inflammatory. However, it is not clear which of these features precludes the other. The aim of this study was to examine the influence of inflammatory cytokines IL-1*β* and IFN*γ* on TDP-43 and p62 in human myotubes and to investigate whether inflammatory conditions can trigger non-inflammatory sIBM-like features. Overall, the results suggest no strong association between IL-1*β* and/or IFN*γ* and TDP-43 or p62 sIBM-like aggregation or TDP-43 mislocalization. However, these results also have broader applicability in understanding the response of muscle cells to inflammation.

Previously, IL-1*β* and IFN*γ* have been used to induce sIBM-relevant non-inflammatory features in human myotubes, causing increased expression of amyloid precursor protein (APP) [26], increased *α*B-crystallin expression [28], increased release of high mobility group box 1 (HMGB1) [29], as well as further inflammatory sIBM-like features of increased inducible nitric oxide synthase and nitrotyrosine [27], increased chemokine and cytokine expression [26], and upregulated inflammasome expression [31]. This shows IL-1*β* and IFN*γ* can influence many processes that have a basis in sIBM pathology; therefore, it was expected these cytokines would dysregulate TDP-43 and p62. The concentration of IFN*γ* used here was higher than that used previously, which may have caused differential effects than lower concentrations. Therefore, a range of concentrations of both cytokines should have been tested. As well as IL-1*β* and IFN*γ*, IFN*γ* and TNF*α* combined treatment has been used to investigate sIBM features in vitro [9, 26, 27, 29], which may be more representative of the sIBM inflammatory milieu as both of these cytokines are secreted by CD8+ T cells, one of the most abundant immune cells infiltrated in sIBM muscle.

IL-1*β* + IFNy treatment increased p62 puncta size, showing promotion of p62 aggregation. However, these puncta were small and not reflective of the large aggregates observed in the sIBM sarcoplasm. p62 aggregation suggests a potential change in autophagy, which was corroborated by an increase in LC3II/LC3I protein. However, this was not accompanied by increased total LC3II, which is observed with increased autophagy [30, 32, 33]. Both increased autophagic flux and blockade of autophagic degradation are accompanied by an increased LC3II/LC3I ratio, which can be elucidated by measuring LC3II expression in the presence of an autophagy blocker such as bafilomycin A1 or chloroquine [30]. Therefore, in the present experiments, it is not clear if autophagy is promoted or blocked with IL-1*β* + IFN*γ* treatment. Increased p62 puncta size was not seen with IL-1*β* or IFN*γ* individually, suggesting activation of multiple inflammatory insults may be necessary to induce p62 puncta alterations.

Despite p62 aggregation being suggested as a marker for sIBM, p62 aggregates are also found in other inflammatory myopathies, most notably autoimmune necrotizing myopathy, as well as polymyositis and dermatomyositis [21, 34]. p62 immunostaining was absent in healthy muscle biopsies but present in patients with inflammatory myopathies as well as in non-inflammatory myopathic conditions, with the accumulation of p62 associated with the severity of muscle damage. The authors concluded that the accumulation of p62 in muscle is a response to muscle injury and not a specific biomarker for sIBM [21]. Increased cytotoxicity was not observed with IL-1*β* + IFN*γ* despite the p62 puncta size increase, showing muscle damage was not the reason for p62 alterations here. IL-1*β* increased p62 protein expression without alteration in LC3II, suggesting increased p62 was not due to autophagic alterations. Instead, this may reflect alterations in pathways other than autophagy. For example, NF-*κ*B, which is downstream of the IL-1 receptor, activates p62 expression [35], and p62 is upregulated in response to cellular stress [36]. The current study did not investigate NF-*κ*B signaling; therefore, it would be interesting to examine NF-*κ*B activity in myotubes with IL-1*β* treatment.

Mislocalization of TDP-43 has been described in sIBM skeletal muscle as well as other conditions such as amyotrophic lateral sclerosis [15, 37–39]. Here, TDP-43 localization was not affected by treatment with IL-1*β*, contradictory to previous experiments with primary rat myotubes using IL-1*β* at the same concentration [9]. The effect of IL-1*β* + IFN*γ* or IFN*γ* on TDP-43 localization in primary human muscle cells has not previously been reported. The analysis here was not restricted to multinucleated myotubes, but as TDP-43 localization was not affected by myotube differentiation (Figure S2), this should not interfere with interpretations. The differences between the results here and those previously published may be due to species-specific differences (human versus rat) or different methods of mislocalization identification. To further test the TDP-43 localization, relative protein expression in subcellular fractions could be examined.

Some cells without cytokine treatment showed TDP-43 localization to the sarcoplasm without clear nuclear expression, suggesting this “mislocalization” can be displayed in healthy myotubes. A study culturing healthy myotubes found approximately 60% of myogenic nuclei contained TDP-43 [40], corroborating the findings here that not all healthy myogenic cells have nuclear TDP-43. This study also compared TDP-43 and p62 aggregates and mislocalization between healthy and sIBM myotubes under electrical stimulation, showing that aberrant TDP-43 and p62 features in sIBM myotubes only arose under culture with electrical stimulation [40], raising the interesting possibility that sIBM-related TDP-43 pathology is linked to muscle contraction/use. Using stimulation conditions for future in vitro sIBM models could be an important consideration.

IL-1*β* and/or IFN*γ* did not consistently affect TDP-43 aggregates or expression. The size of the TDP-43 aggregates was small and observable in the control conditions, so they may be performing homeostatic functions such as myogenic RNA processing [41]. As p62 was susceptible to cytokine treatments while TDP-43 was not, this suggests TDP-43 and p62 are not similarly regulated by inflammatory conditions in myotubes. To further examine TDP-43 expression under cytokine conditions, the expression in the insoluble protein fraction could be examined, as this would better reflect the aggregated state of TDP-43.

As an alternative to the hypothesis tested here, it is possible that noninflammatory features of sIBM, including TDP-43 and p62-containing inclusion bodies, can trigger inflammation. In fact, TDP-43 overexpression in astrocytes caused increased secretion of proinflammatory cytokines, including IL-1*β*, IL-6, and TNF*α* [42]. Exposure of microglia to extracellular TDP-43 aggregates leads to IL-1*β* secretion [43], and similarly, extracellular exposure of microglia to wild-type, truncated, or mutant TDP-43 activates microglia and upregulates TNF*α* and IL-1*β* expression [44]. Investigating TDP-43 overexpression or extracellular aggregates in myotubes would shed light on whether non-inflammatory myositis features can trigger muscle inflammation, for which there is evidence for the sIBM-related protein *β* APP [9]. Furthermore, overexpression of *β* APP in myotubes triggered TDP-43 aggregates and mislocalization [9], showing non-inflammatory sIBM-like features can themselves trigger further non-inflammatory features. This suggests inflammation may not be necessary for the presence of non-inflammatory s-IBM-like features.

## 5. Conclusions

Overall, the addition of IL-1*β* and/or IFN*γ* at the concentrations tested failed to recapitulate the sIBM-like phenotype of sarcoplasmic TDP-43 and p62 aggregation. Although the size of p62 puncta increased with IL-1*β* + IFN*γ* treatment, this was not accompanied by increased protein expression and was likely due to autophagic changes. This suggests treating myotubes with IL-1*β* + IFN*γ* may not trigger some non-inflammatory sIBM-like features. IL-1*β* increased expression of the p62 protein without influencing p62 puncta, most likely through an autophagy-independent mechanism. The lack of effect on TDP-43, along with some effects observed with p62, along with the lack of colocalization suggests differential regulation of these two proteins under exposure to inflammatory cytokines. There were limitations to this study due to the short time period of IL-1*β* and IFN*γ* exposure considering the progressive, chronic nature of myositis conditions, as well as a lack of investigation of different IL-1*β* and IFN*γ* concentrations.

## Data Availability

Data supporting this study are included within this article and/or supporting materials. Full data are available on request from the authors.

## Supplementary Materials

Supplementary Materials Table S1: antibodies and their dilutions. Figure S1: increase in TDP-43 aggregate size in 3/5 myogenic donors. Figure S2: TDP-43 localisation and expression during myogenesis. Figure S3: muscle cell donor characteristics. Table S2: comparison of control conditions between the two sets of experiments with IL-1*β* + IFN*γ*, or IL-1*β* or IFN*γ*. Table S3: list of myogenic donors used in each experiment.
