# Supplementary material for: Influence of Inflammatory Cytokines IL-1β and IFNγ on Sarcoplasmic Aggregation of p62 and TDP-43 in Myotubes
Source: Mediators Inflamm. 2023 Sep 12;2023:9018470. doi: 10.1155/2023/9018470 (PMC10509004; doi:10.1155/2023/9018470)
Supplement: Supplementary Materials — Table S1: antibodies and their dilutions. Figure S1: increase in TDP-43 aggregate size in 3/5 myogenic donors. Figure S2: TDP-43 localisation and expression during myogenesis. Figure S3: muscle cell donor characteristics. Table S2: comparison of control conditions between the two sets of experiments with IL-1β + IFNγ, or IL-1β or IFNγ. Table S3: list of myogenic donors used in each experiment. [file 9018470.f1.docx]

**Supplementary material**


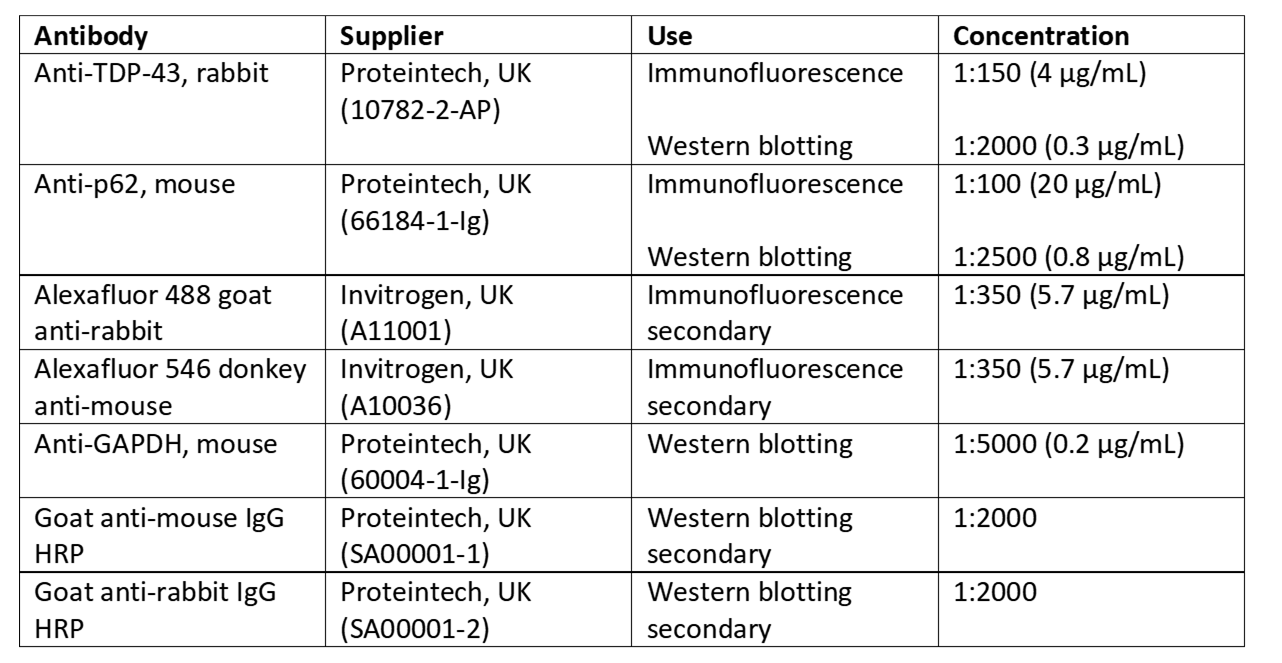


Table S1. Antibodies and their dilutions.


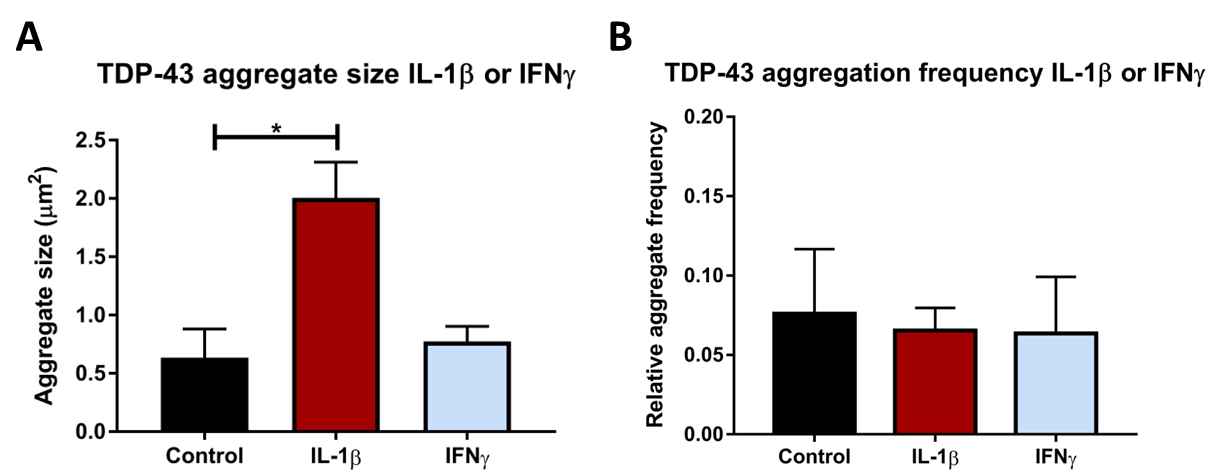


Figure S1. Increase in TDP-43 aggregate size in 3/5 myogenic donors. There was a significant increase in TDP-43 size (p = 0.0119) with IL-1β treatment compared to control, without a change in relative TDP-43 aggregate frequency. n = 3 myogenic donors, One-way ANOVA with Dunnett’s multiple comparisons.


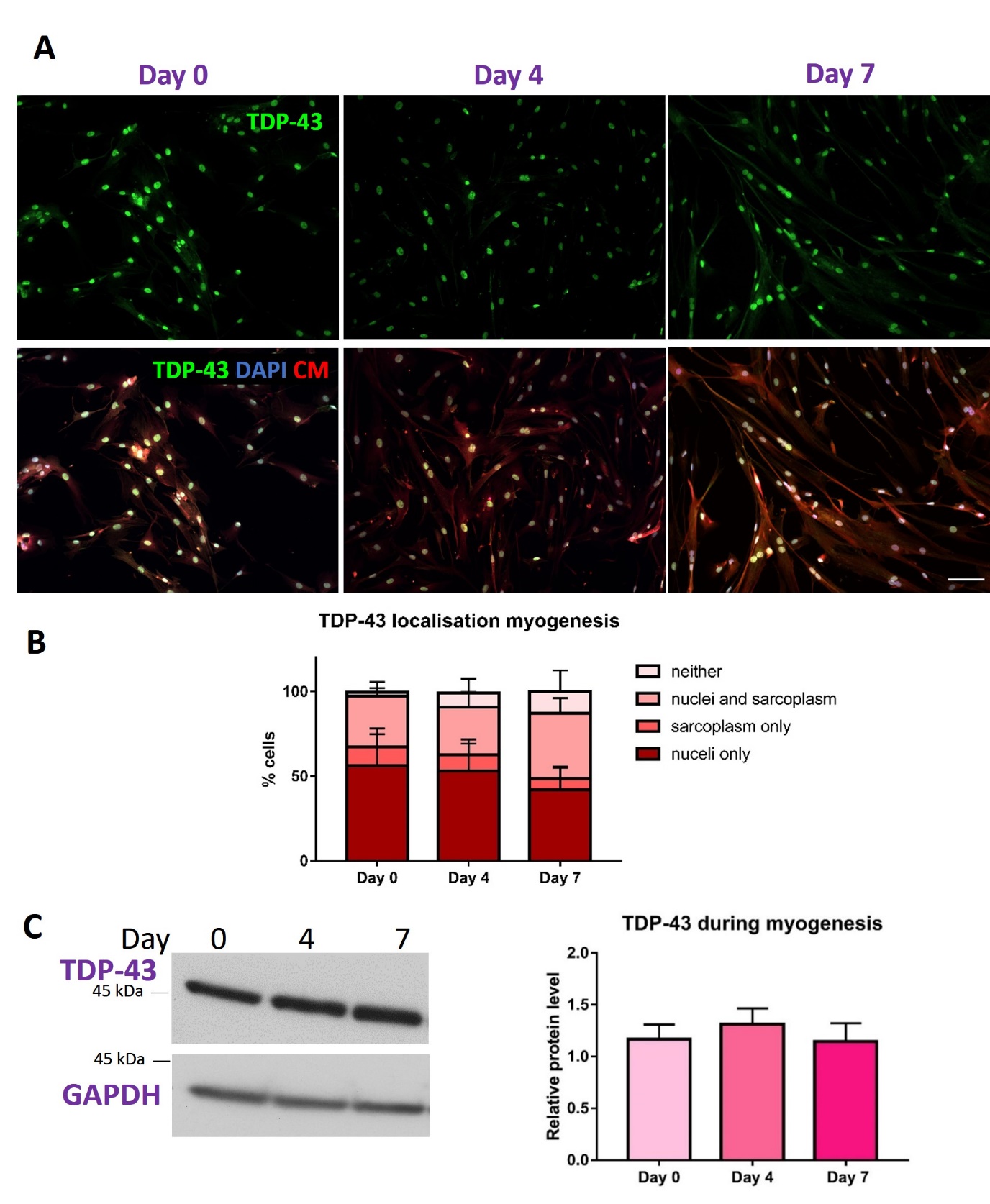


Figure S2. TDP-43 localisation and expression during myogenesis. TDP-43 was analysed on day 0 (proliferation of myoblasts), and days 4 and 7 of differentiation. (A) Images of TDP-43 distribution on day 0, 4, and 7 of differentiation. CM- cell mask. Scale bar = 100 µm. (B) TDP-43 distribution n = 4 donors. There was no difference in TDP-43 localisation between timepoints (two-way ANOVA). (C) Total TDP-43 expression levels during myogenesis normalised to GAPDH. There was no difference in expression between time points (One-way ANOVA, n = 6 myogenic donors).


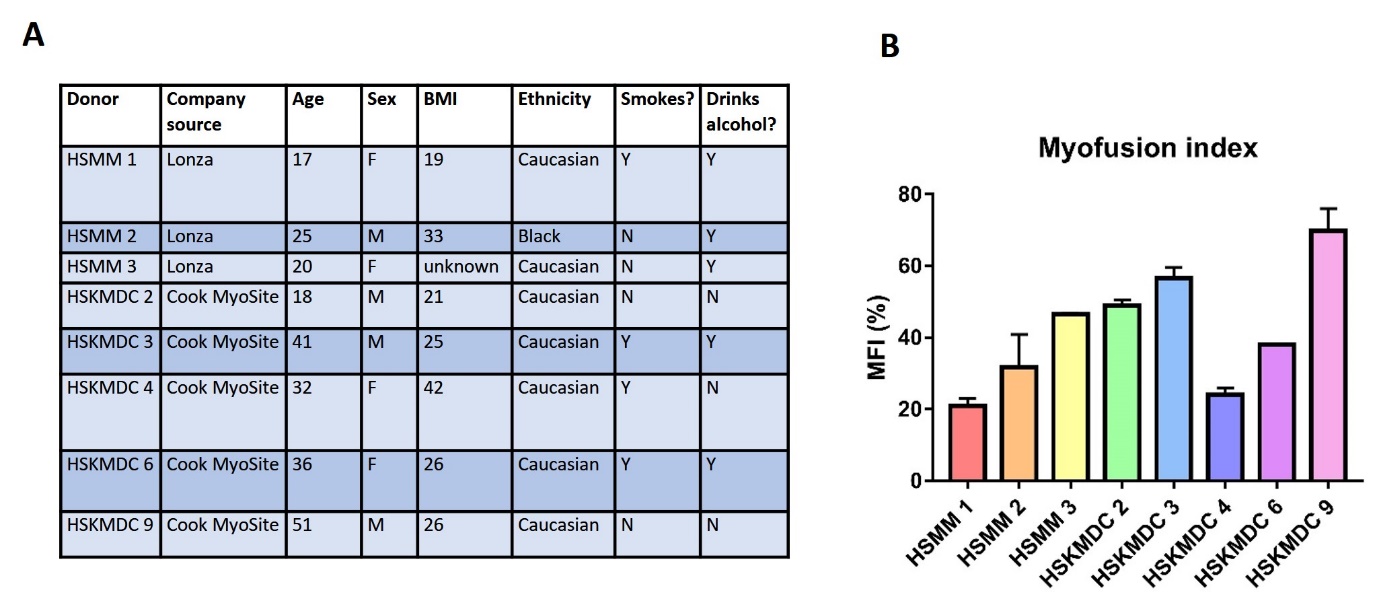


Figure S3. Muscle cell donor characteristics. (A) Table showing the demographic characteristics of commercially sourced muscle cells (B) Myofusion index (MFI) of the donors after 9 days of differentiation. MFI is the percentage of nuclei within a myotube compared to the total number of nuclei. n = 2 biological repeats.


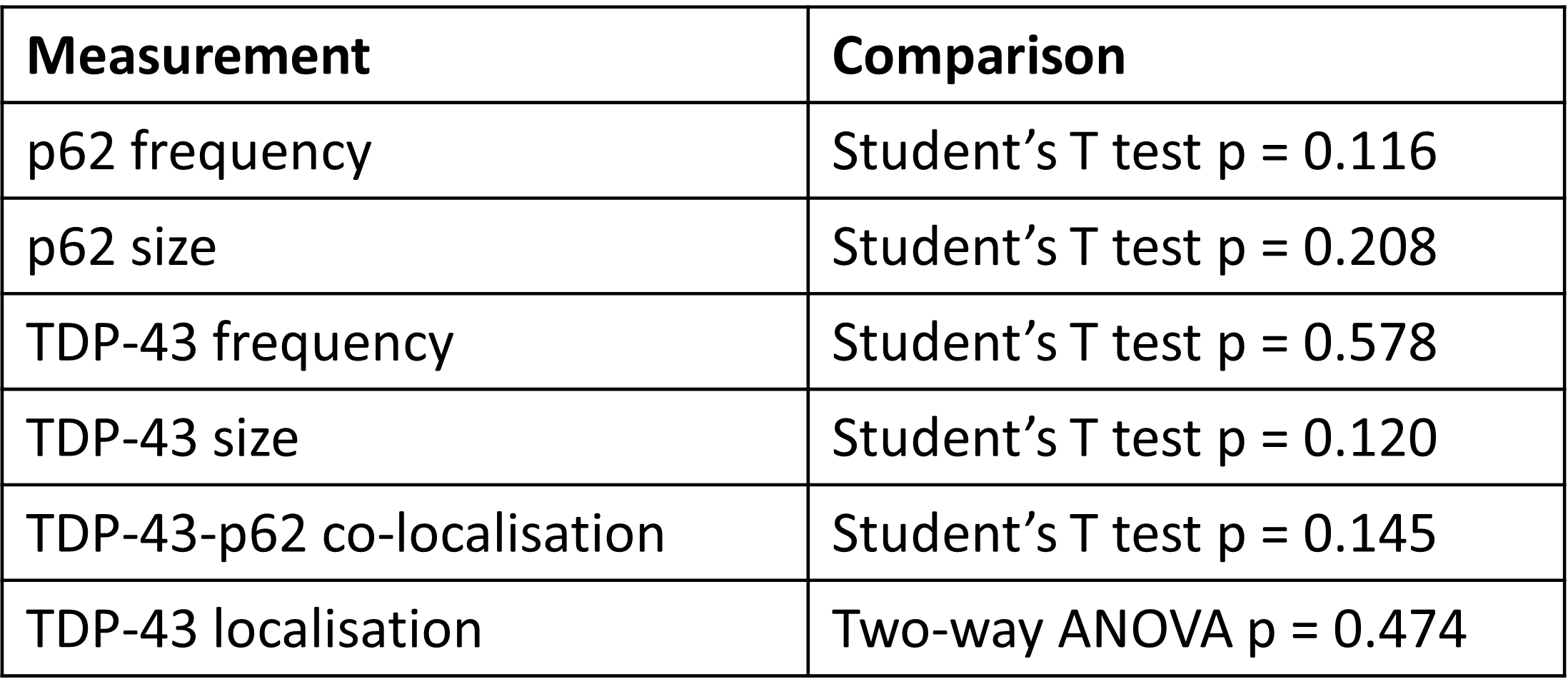


Table S2. Comparison of control conditions between the two sets of experiments with IL-1β+IFNγ, or IL-1β or IFNγ. All Student’s T test comparisons passed Shapiro-Wilk normality testing.


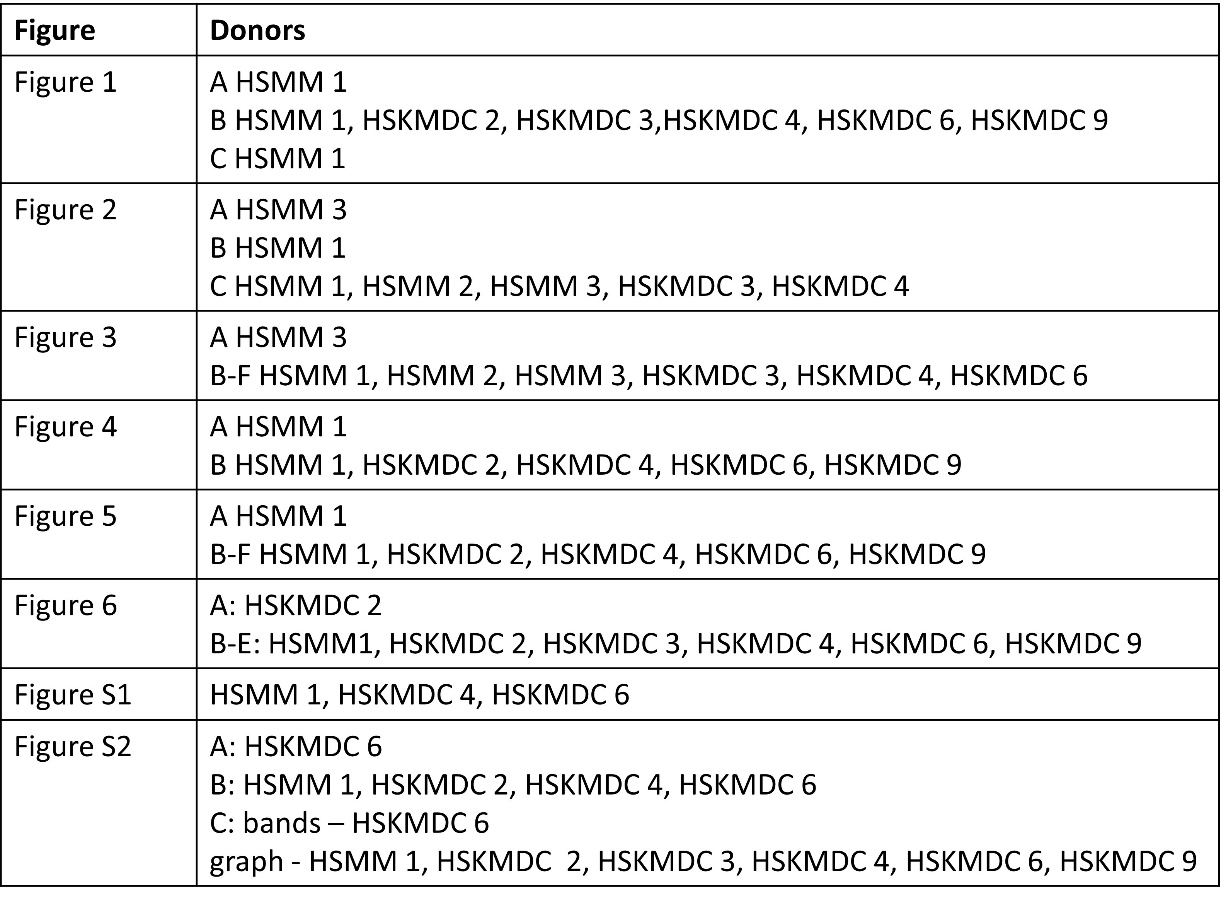


Table S3. List of myogenic donors used in each experiment.
